# Exploring Prey Selectivity and Feeding Habits of Wels Catfish (*Silurus glanis* L., 1758) in a Deep Anatolian Reservoir: Seasonal, Length, and Age-Dependent Diet Analysis

**DOI:** 10.1155/anu/4619857

**Published:** 2025-04-24

**Authors:** Ramazan Yazici, Mahmut Yilmaz, Okan Yazicioğlu

**Affiliations:** ^1^Laboratory and Veterinary Health Program, Veterinary Department, Çiçekdağı Vocational School, Kırşehir Ahi Evran University, Kırşehir, Türkiye; ^2^Agricultural Biotechnology Department, Faculty of Agriculture, Kırşehir Ahi Evran University, Kırşehir, Türkiye; ^3^Department of Plant and Animal Production, Vocational School of Technical Sciences, Kırşehir Ahi Evran University, Kırşehir, Türkiye

**Keywords:** diet content analysis, feeding features, food, prey preference, Sıddıklı dam lake, wels catfish

## Abstract

Feeding habits and dietary preferences of wels catfish (*Silurus glanis*) were investigated in Sıddıklı Dam Lake through the examination of 200 individuals. The results revealed that the species predominantly exhibited piscivorous feeding characteristics, with *Tinca tinca* (IRI% = 78.93) identified as the primary food source. The food items in the stomach showed a wide spectrum, ranging from benthic invertebrates, crustaceans, molluscs, amphibians, and mammals to fishes. The study not only assessed the general food composition of wels catfish but also delved into the seasonal variations in diet composition. It was found that the stomach fullness index (FI) varied significantly among the seasons, with Winter showing the highest values (0.827). On the other hand, the lowest value was detected in the Autumn season (0.480). Age and length groups were also considered, with notable differences in stomach FI and diet composition observed across different stages of growth. Food preference analysis highlighted the selective tendencies of wels catfish towards certain food types, with *Atherina boyeri* and *T. tinca* emerging as preferred choices in different size groups. For small, medium, and large length individuals, the most preferred prey fish were *A. boyeri* (Va = 0.39518, *χ*2 = 31.2336), *T. tinca* (Va = 0.63564, *χ*2 = 82.8073) and *T. tinca* (Va = 0.666495, *χ*2 = 88.4307), respectively. The findings provide valuable insights into the feeding behaviour of wels catfish, underscoring the importance of understanding these patterns for effective management and conservation efforts. Further research should aim to explore the ecological implications of these feeding habits on the overall aquatic ecosystem.

## 1. Introduction

The wels catfish (*Silurus glanis* L., 1758) is a fish species with high economic value. It has been cultivated in many countries for many years [1–3]. This species is distributed in the North Sea, Baltic Sea, Caspian Sea, Black Sea and Aral Sea basins, the northern regions of Sweden and Finland, the Aegean Sea basin, and Türkiye [4]. Moreover, in recent years, it has been reported to have spread from Western Europe to England. Wels catfish can typically grow up to 2–3 m in length and weigh more than 130 kg [5]. Although it has an omnivorous feeding habit, it is predominantly predatory, and its food spectrum is quite broad. Young individuals generally feed on insect larvae, small crustaceans, and fish fry, while adults prey on fish, birds, amphibians, rodents, and other small vertebrates [6, 7].

The feeding habits of the wels catfish make it an important predator in the ecosystem. As a carnivore, it can prey on various fish species, crustaceans, amphibians, and even small mammals [8]. Nocturnal by nature, this fish uses its strong sense of smell to effectively locate and capture prey [9]. Its feeding habits are crucial not only for individual survival and growth but also for influencing the population dynamics of the prey species and maintaining ecological balance [10]. Understanding the predatory behaviors and diet composition of the wels catfish is critical for comprehending its role in the ecosystem.

From a fisheries biology perspective, understanding the feeding characteristics of the wels catfish (*S. glanis*) is essential for multiple reasons. First and foremost, understanding the impact of this species on prey fish populations plays a crucial role in managing aquatic resources and developing conservation strategies [7, 11]. *S. glanis* significantly influences regional ecosystem dynamics by affecting predator–prey relationships and biological diversity. Therefore, a detailed examination of the wels catfish's feeding behaviors not only enhances scientific knowledge but is also vital for the sustainable management of freshwater ecosystems [12, 13].

Fish size, condition, and feeding habits are influenced by various factors, including food availability, habitat type, age, location, and seasons [14]. The food web in aquatic ecosystems can be studied through fish stomach contents and other ecological indicators [14, 15]. Insights into the natural feeding behaviors of wels catfish also provide essential information for aquaculture practices. Determining suitable diet types in aquaculture is crucial for optimizing the growth performance and health of this species. Effective feeding strategies promote sustainable fish production, economic efficiency, and minimized ecosystem impact [16, 17].

Research focusing on the biology and ecosystem role of the wels catfish (*S. glanis*) facilitates the understanding of biological control mechanisms and makes significant contributions to conservation biology [7, 18]. Analyzing this species' diet, habitat preferences, and reproductive strategies provides essential data for the sustainable management of water resources and the formulation of fisheries policies [11]. Therefore, this study aims to understand the effects of this species on aquatic ecosystems by examining the food items and feeding habits of the *S. glanis* species in detail and contribute to the development of sustainable fisheries management and conservation strategies.

## 2. Materials and Methods

### 2.1. Fish Sampling and Laboratory Processes

Wels catfish samples were caught monthly from Sıddıklı Dam Lake between September 2015 and August 2016. Trammel and gill nets were used to capture fish. As a result, a total of 200 *S. glanis* samples were obtained. The total and standard lengths of the samples were measured with an accuracy of ± 1 mm, and their weights were weighed with an accuracy of ± 0.01 g.

The digestive system of wels catfish, from the esophagus to the anus, was cut with scissors and preserved in a 4% formaldehyde solution [19, 20, 21]. The samples were cleared of fat, mesentery fragments, and other foreign matter and tissue fragments [22].

The stomach samples that were ready for examination were weighed using a scale with a precision of ± 0.01 g, and the food types in the stomach contents were grouped to be identified. The weight values of each food group and empty stomachs were weighed with an accuracy of ± 0.01 g. Identification of macro-level organisms in the stomach content was made at the lowest possible taxonomic level using a binocular microscope. Various sources were used during these diagnoses [23, 24].

### 2.2. General Diet Analysis

In the analysis of the stomach contents to determine which food types *S. glanis* consumed, their abundance and importance, numerical percentage (*N*%), weight percentage (*W*%), and frequency of occurrence (FO%) methods were used [25, 26, 19, 22].

  $$\begin{array}{c} FO\text{\% }=\frac{\text{number of stomachs containing prey }i}{\text{Number of stomach with any food item}}\times 100 \end{array}$$

  $$\begin{array}{c} N\text{\% }=\frac{\text{Total number of prey }i}{\text{Total number of all prey items}}\times 100 \end{array}$$

  $$\begin{array}{c} W\text{\% }=\frac{\text{Total weight of prey }i}{\text{Total weight of all fprey items}}\times 100 \end{array}$$

Fullness index (FI) and vacuity index (VI) methods were used to detect changes in the feeding intensity of wels catfish [25, 27–29].

  $$\begin{array}{c} FI\text{\% }=\frac{\text{Total weight of stomach contents}}{\text{The weight of fish}}\times 100 \end{array}$$

  $$\begin{array}{c} VI\text{\% }=\frac{\text{Number of empty stomachs}}{\text{Total number of stomachs examined}}\times 100 \end{array}$$

In order to determine the importance of food types in the *S. glanis* sample, the index of relative importance (IRI), which is the synthesis of numerical percentage (*N*%), percentage weight (*W*%) and frequency of occurrence (FO%) methods, was used. This index was calculated with the following formula [30, 31].

  $$\begin{array}{c} IRI=\left(N\text{\% }+W\text{\% }\right)\times FO\text{\% } \end{array}$$

The above equation was modified by Hacunda [32] and IRI% values were calculated according to the equation below.

  $$\begin{array}{c} IRI\text{\% }=\left(\text{IRI}/\sum \text{IRI}\right)\times 100 \end{array}$$

### 2.3. Seasonal Analysis

The seasonal diet analyses were made separately Winter (December, January, and February), Spring (March, April, and May), Summer (June, July, and August) and Autumn (September, October, and November). To analyze the seasonal pattern, numerical percentage (*N*%), weight percentage (*W*%), frequency of occurrence (FO%), and index relative importance percentage (IRI%) were used. The overlap (similarity) in the food composition of wels catfish according to seasons was determined by Schoener's Overlap Index (*C*_xy_) [33]. Schoener's Overlap Index value varies between 0 and 1. If this value is greater than 0.60, it means that the food of the two groups is similar, and if it is smaller, it means that there is no similarity between the food of the two groups [34].

### 2.4. Analysis by Age Groups

Stomach content analyzes of wels catfish were examined separately according to age. Age data was obtained from [35]. The ages of the samples ranged from 1 to 11 years, and there were samples for each age group. FI% and VI% were used to determine how the rates of full and empty stomachs changed according to age groups. Additionally, IRI% was used to determine the pattern of importance of food types present in the species' diet with age.

### 2.5. Analysis by Length Groups

The total lengths of individuals ranged from 20.41 to 101.0 cm. In order to make the analyses according to length understandable, the samples were classified as small (20.41–47.0 cm), medium (47.1–74.0 cm), and large (74.1–101.0 cm) sized individuals. FI% and VI% were used to assess how the proportions of full and empty stomachs varied across different length groups. Additionally, IRI% was utilized to identify the pattern of importance of different food types in the species' diet in relation to length. The similarity of food composition of *S. glanis* according to length groups was determined using Schoener's Overlap Index (*C*_xy_) [33].

### 2.6. Prey Selection

Pearre's Selective Index (*V*_a_) was used to determine the relationship between fish size and the food consumed in the sample, which was divided into small, medium, and large length groups, and to identify any selection present. This index takes a value between + 1 (strong positive selection) and −1 (strong negative selection) when 0 is considered neutral [36, 37]. The relative abundances of fish species in the habitat required to calculate Pearson's selectivity index were obtained from [2, 3]. The densities of existing fish species in the Sıddıklı dam lake are *Atherina boyeri* 43.6%, *Cyprinus carpio* 14.48%, *Tinca tinca* 13.64%, *S. glanis* 11.95%, *Squalius seyhanensis* 8.43%, *Esox lucius* 6.76%, *Alburnus escherichii* 0.85%, and *Capoeta tinca* 0.29%. The significance of the selectivity index was evaluated using the chi-square test:

  $$\begin{array}{c} \chi^{2}=n\times V_{a}^{2}, \end{array}$$

where *n* is the number of samples.

## 3. Results

### 3.1. General Food Composition

The digestive tract contents of 200 wels catfish caught from Sıddıklı Dam Lake were examined. It was determined that 47% (*N* = 94) of the individuals examined had an empty stomach and 53% (*N* = 106) had a full stomach. Numerical percentage (*N*%), percentage weight (*W*%), frequency of occurrence (FO%), IRI and percentage value (IRI%) of food types are presented in Table 1.

A total of 18 food types belonging to fish, amphibian, mammal, mollusca, crustacean, and benthic invertebrate groups were detected in the stomach contents of wels catfish. The main food of the species is *T. tinca* (IRI% = 78.9) from fish groups. When the general food composition was examined, it was determined that the species showed piscivorous feeding characteristics. Additionally, no cannibalism was observed in the sample.

### 3.2. Seasonal Feeding Characteristics

**Figure 1:**
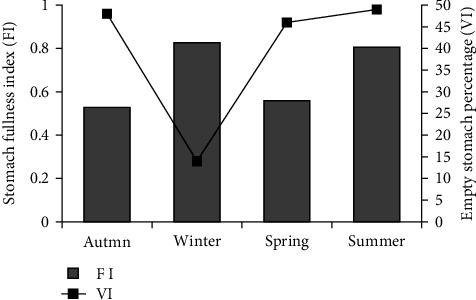
Seasonal change in stomach fullness index and empty stomach percentage.

Stomach FI, which is an indicator of feeding activity, varied between seasons. The average stomach FI reached its highest value (0.827) in the Winter. On the other hand, the lowest value (0.480) was determined in the Autumn. When empty stomach percentages are examined, the lowest percentage of empty stomach was recorded in Winter (14.3%), and the highest value was recorded in Summer (48.9%) (Figure 1).

It was determined that the most important food item for wels catfish in Spring is *T. tinca* (IRI% = 67.74). This was followed by fish remains (IRI% = 9.9%) and *C. carpio* (IRI% = 7.1) food types. Additionally, Ranidae (IRI% = 6.1), *A. boyeri* (IRI% = 5.5), unidentified fish (IRI% = 2.5), Isopoda (IRI% = 1.1), and *Astacus spp*. (IRI% = 0.4) are other foods consumed (Table 2).

In Summer, *T. tinca* (IRI% = 75.84) constituted the main food item of the species. *A. boyeri* (IRI% = 14.3) was determined as the second important food type. Additionally, fish remains IRI% = 2.62), *Astacus spp*. IRI% = 1.94), Ranidae IRI% = 1.57), *Potamon spp*. IRI% = 1.46), unidentified fish IRI% = 0.73), Dipter larva IRI% = 0.3), *Squalius seyhanensis* IRI% = 0.29), *C. carpio* IRI% = 0.23), *Gammarus spp*. IRI% = 0.11), *A. escherichii* IRI% = 0.11), Dipter pupa IRI% = 0.08), Odonata IRI% = 0.032), Bivalvia IRI% = 0.03), and *S. glanis* (IRI% = 0.028) consumed (Table 2).

*T. tinca* (IRI% = 83.9) was determined as the most consumed food type in the Autumn season. This was followed by fish remains (IRI% = 8.4) and unidentified fish (IRI% = 6.4) groups. In addition, it was determined that the importance of Rodentia (IRI% = 1.1) and *C. carpio* (IRI% = 0.2) foods in the diet was quite low (Table 2).

*T. tinca* (IRI% = 63.0) constituted the main food item of wels catfish in Winter. Fish remains (IRI% = 15.6) were identified as the second important nutrient. In addition, Rodentia (IRI% = 10.7), unidentified fish (IRI% = 8.4) and *C. carpio* (IRI% = 2.3) were consumed at low rates (Table 2).

According to Schoener overlap index (*C*_xy_) values, it was determined that the diets of wels catfish were similar in all seasons except Summer and Winter (*C*_xy_ = 0.596251). The highest similarity was detected in the Autumn and Winter diets (*C*_xy_ = 0.936877) (Table 3).

### 3.3. Feeding Features According to Age Groups

**Figure 2:**
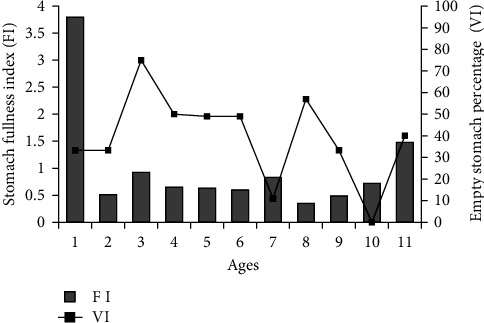
Changes in stomach fullness index and empty stomach percentage according to age.

It has been determined that the stomach FI varies between age groups. It was determined that the average stomach FI value was highest (3.8) in 1-year-old fish and lowest (0.23) in 3-year-old fish. When empty stomach percentages are examined, the lowest empty stomach percentage was found in 10-year-old fish (0.0%), and the highest empty stomach percentage was found in 3-year-old fish (75%). Both stomach FI and empty stomach percentage generally tended to decrease with advancing age (Figure 2).

When the stomach contents are examined in terms of age groups, the main food of 1-, 2-, and 3-year-old wels catfish is *A. boyeri* (IRI%>75). In the sample, the primary food of individuals aged 4, 5, 6, 7, 9, 10, and 11 years was *T. tinca* (4th age IRI% = 91.3, 5th age IRI% = 80.4, 6th age IRI% = 85.9, 7th age IRI% = 47.2, 9th year IRI% = 81.1, 10th year IRI% = 80.2, 11th year IRI% = 53.8). However, in 8-year-old fish, the IRI% value of *T. tinca* decreased to 28.1 and the IRI% value of Rodentia increased to 26.8, and these two species are considered the main food in this age group (Table 4).

In general, *T. tinca* was consumed extensively by fish aged 4 years and above but was not consumed by individuals aged 1, 2 and 3 years. Depending on the increase in age, a decrease in the importance of Odonata, Bivalvia, Isopoda, *Gammarus spp*., dipter pupae, and dipter larvae in the diet was determined. It has also been determined that wels catfish feed entirely on fish and vertebrates after the age of 4. Finally, the importance of *C. carpio* in the diet, which has a higher body size than other forage fish, increased at the ages of 10 and 11 (Table 4).

### 3.4. Feeding Features According to Length Groups

**Figure 3:**
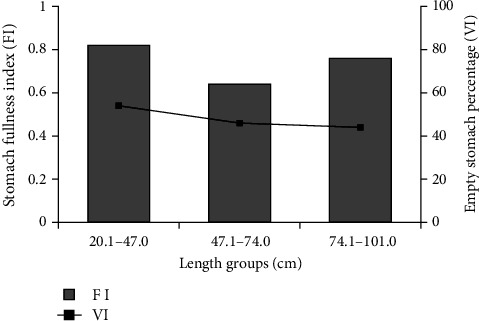
Changes in stomach fullness index and empty stomach percentage according to length groups.

Stomach FI varied between length groups. It was determined that the average stomach FI was at the highest level (0.82) in small-sized (<47.1 cm) individuals, while it was at the lowest level (0.64) in medium-sized (47.1–74.0 cm) individuals. The lowest value of empty stomach percentage (44%) was found in individuals with large length (>74.1 cm), while the highest value (54%) was found in the small length group (<47.1 cm). The most intense feeding activity was detected in samples from the small-size group (Figure 3).

When stomach contents were examined according to size groups, *A. boyeri* (IRI% = 73.3) constituted the most important food of small-sized wels catfish. The secondary important food type was *T. Tinca* with an IRI value of 15.7%. The main food type of wels catfish in both medium and large size groups was determined as *T. tinca* (medium size, IRI% = 83.8; large size, IRI% = 68.2) (Table 5).

Benthic invertebrates were consumed mostly by small-sized wels catfish. However, they are also consumed, to a small extent, by medium-sized wels catfish. In addition, the type of food consumed in small-sized wels catfish was less diverse compared to other size groups. Common carp have never been consumed by small-sized individuals, and their importance in the diet increases as the size increases. Food diversity in medium-sized wels catfish increased and all fish species present in the Sıddıklı dam lake were included in the food composition. Ranidae group food species are included in the diet of medium-sized wels catfish and have increased their importance in the diet of large-sized wels catfish. *Astacus spp*. was consumed more by small-sized wels catfish and had no effect on the diet in the medium and large-sized wels catfish. Large-sized wels catfish have a completely vertebrate diet and mostly fish are consumed as a food type (Table 5).

According to Schoener overlap index (*C*_*xy*_) values, similarity was detected only in the diets of medium- and large-sized wels catfish (*C*_*xy*_ = 0.8534). As a result of other pairwise comparisons between length groups, no similarity (*C* < 0.60) was determined in terms of food consumed (Table 6).

### 3.5. Food Preference of Wels Catfish

**Figure 4:**
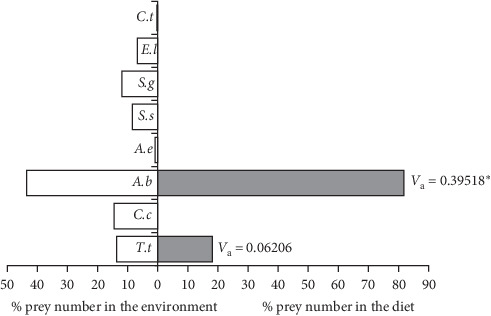
Pearre's selectivity index values in small-sized wels catfish.

When Pearre's Selectivity index values were examined in small-sized (20.1–47.0 cm) samples, *A. boyeri* (*V*_a_ = 0.39518) was the most preferred food type by individuals in this length group, and the selectivity index value was statistically significant (*x*^2^ = 31.2336, *p*  < 0.001). Similarly, *T. tinca* (*V*_a_ = 0.062060975) was selected as positive, but the selectivity index value was not statistically significant (*x*^2^ = 0.77031, *p*  > 0.05). No other fish species were consumed as food by individuals of the small-size group. For this reason, the selectivity index values of other species were accepted as 0, and it was concluded that they were not selected in any way (Figure 4).

**Figure 5:**
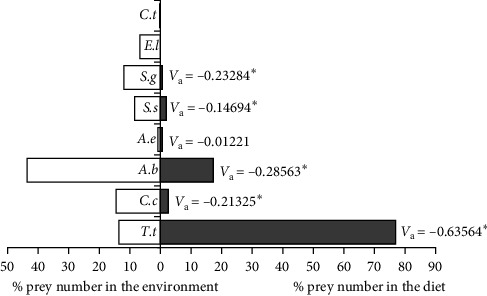
Pearre's selectivity index values in medium-sized wels catfish.

When the selectivity index values in individuals in the medium size (47.1–74.0 cm) group were examined, *T. tinca*, whose density was relatively low in the lake, was positively selected and this selectivity was determined to be statistically significant (*V*_a_ = 0.63564, *x*^2^ = 80.8073, *p*  < 0.001). On the other hand, *C. carpio* (*V*_a_ = −0.21325, *x*^2^ = 9.09487, *p*  < 0.005), *A. boyeri* (*V*_a_ = −0.28563, *x*^2^ = 16.3165, *p*  < 0.001), *S. glanis* (*V*_a_ = −0.232837732, *x*^2^ = 10.84268185, *p*  < 0.001), and *S. seyhanensis* (*V*_a_ = −0.14694, *x*^2^ = 4.311816, *p*  < 0.025) species were selected negatively, and these selections are also statistically significant. Similarly, *A. escherichii* (*V*_a_ = −0.01221, *x*^2^ = 0.02982, *p*  > 0.05) was negatively selected by wels catfish, but it was determined that this situation did not have any statistical significance (Figure 5).

**Figure 6:**
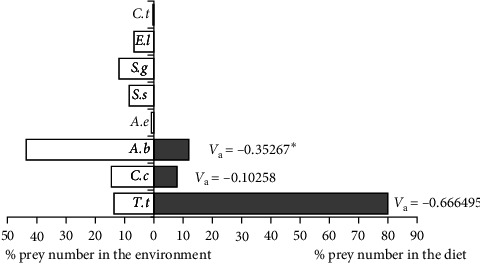
Pearre's selectivity index values in large-sized wels catfish.

Large-sized (74.1–101.0 cm) wels catfish positively selected the *T. tinca* (*V*_a_ = 0.666495, *x*^2^ = 88.4307, *p*  < 0.001) species, which they consume as their main food, and this trend was statistically significant. *A. boyeri* (*V*_a_ = −0.35267, *x*^2^ = 24.8749, *p*  < 0.001), the most abundant species of the Sıddıklı dam lake, was significantly negatively selected by *S. glanis* individuals. Similarly, *C. carpio* (*V*_a_ = −0.10258, *x*^2^ = 2.10444, *p*  > 0.05) was also selected as negative, but this trend was not statistically significant (Figure 6).

## 4. Discussion

In this study, the empty stomach rate of wels catfish was determined to be 47%. The empty stomach rate of the species was reported as 43.83% in Hirfanlı Dam Lake [38], 45.0% in Feldberger Haussee Lake [39], and 50.8% in Menzelet Dam Lake [40]. In general, it is seen that the empty stomach rate in wels catfish is around 50%. The percentage of empty or full stomachs may vary depending on many factors such as the fish's waiting time in the net, sampling time, distribution of sampling density according to months and seasons, length distribution, nutritional capacity of the habitat, environmental parameters, and fishing methods. In addition, wels catfish have piscivorous feeding characteristics and are active hunters at night, which explains the high empty stomach rate. As a matter of fact, Vinson and Angradi [41] reported in their study that piscivorous fish have a higher percentage of empty stomachs than others, and similarly, the same situation applies to nocturnal predators. In addition, Arrington et al. [42] reported that the empty stomach rate of the Siluridae family may be generally higher than other fish species.

Wels Catfish were fed mostly with fish species (*T. tinca*, *C. carpio*, *A. boyeri*, *A. escherichii*, *Squalius seyhanensis*, *S. glanis*, and unidentified fish). In addition, it also consumed mammals, crustaceans, molluscs, amphibians, and benthic invertebrates as food. The main food of the species is *T. tinca* (IRI% = 78.9).

The food types consumed by wels catfish living in different habitats are presented in Table 7. In this study, it is understood that the species has piscivorous feeding characteristics according to its general food composition. According to the results of studies conducted by other researchers, it is seen that the feeding of the wels catfish in many different habitats is mostly based on fish species [8, 38, 40, 43–51]. On the other hand, a small number of food types from other vertebrates, invertebrates, and crustaceans have been reported in the diet of wels catfish (Table 7). It is thought that the higher consumption of fish species in the study is due to the size distribution of the studied samples and the larger body size of food fish compared to benthic invertebrates. It is understood that the food range of wels catfish is very wide. In this study, a total of 18 food types belonging to six groups were consumed, and it was determined that they had a wide range of foods. As a matter of fact, a similar situation was reported by Copp et al. [7]. Wels catfish are also described as opportunistic [52] and scavenger or forager [53] predators. It is thought that the richness in its nutritional composition may be due to the fact that it is an opportunistic and foraging species. It is also thought that the fact that this species consumes many types of food may be due to food scarcity or low energy values of foods. On the other hand, food diversity varies according to habitats. Pavlović et al. [51] examined the stomach contents of wels catfish and reported that species belonging to the Cyprinidae family constituted the nutritional composition. Additionally, some researchers have emphasized the importance of crayfish species in the diet of wels catfish [8, 49, 54].

No cannibalism phenomenon was encountered in this study. The lack of cannibalism may be due to factors such as the abundance of species belonging to the Cyprinidae family in the lake and wels catfish having a wide spectrum of food types. In some studies, it has been reported that individuals of their own species are found in the stomach contents of wels catfish, but this rate is too low to be considered cannibalism [38, 40].

### 4.1. Seasonal Feeding Characteristics

As a result of examining the empty stomach rate according to seasons, it was found that it was highest in Summer (48.9%) and lowest in Winter (14.3%). It is thought that the high percentage of empty stomach in Summer is due to the rapid digestion of food as a result of the increase in metabolic rate in parallel with the increase in temperature. An increase in the empty stomach rate started from the Spring season and reached its highest level in the Summer season. During these seasons, which include the breeding period of the species, feeding may have slowed down due to reproductive activities. It is thought that the decrease in the empty stomach rate in Winter is due to the acceleration of feeding activities in the prebreeding period and the length of the dark period in this season, as it is a nocturnal hunting species. Long night hours increase the probability of wels catfish, which are nocturnal hunters, to find food and therefore cause the empty stomach rate to be low. Like the findings of this study, Alp [40] found the highest percentage of empty stomachs in the study he conducted in Menzelet Dam Lake in the Spring season, which is the breeding season of the species.

There are differences in the food types of wels catfish depending on the seasons. However, the main food type did not differ between seasons. There is richness in terms of food diversity in the Spring and Summer seasons. However, diversity decreased significantly in the Winter and Autumn seasons. As a matter of fact, Doğan Bora and Gül [38] reported in their study in Hirfanlı Dam Lake that the food diversity of wels catfish reached its highest value in the Summer season, whereas there was a narrowing in the food range in the Autumn and Winter seasons. In his research conducted in Menzelet Dam Lake, Alp [40] reported that the food diversity in the stomach contents of wels catfish was more intense in the Spring and Summer seasons compared to the Autumn and Winter seasons. The findings of this study are compatible with studies in different habitats in terms of seasonal feeding density. Since the density of the foods consumed by the species in habitats varies seasonally, it is normal to see food diversity in their stomach contents.

Özdemir [59] stated that with the decrease in water temperature, species need less feeding. In this case, the decrease in food diversity may be related to this phenomenon, as the species that make up the food of wels catfish will also engage in less foraging activities. Also, fish species breeding, etc. Migration in different seasons for many reasons also contributes to this situation. Many species present in the Spring and Summer diet are not consumed in other seasons. In particular, freshwater crayfish were not consumed in the Winter and Autumn diets. Similarly, Czarnecki et al. [54] reported in their study in Lake Goreckie that crayfish species were consumed abundantly in the Summer and Spring diets of wels catfish but were not included in the Winter and Autumn diets.

Rodentia consumed in Autumn and Winter seasons was not consumed in other seasons. This situation can be explained by the decrease in the abundance of fish species in the Autumn and Winter seasons and the wels catfish experiencing food shortages and therefore turning to food types that are not available in other seasons.

### 4.2. Feeding Features According to Age Groups

According to age groups, *T. tinca* was the main food for wels catfish aged 4 and above, while *A. boyeri* was consumed as the main food for 1, 2 and 3 year olds. The importance of benthic invertebrates in the diet decreased with increasing age. It has been determined that wels catfish only prey on fish, crustaceans, and other vertebrates after the age of 4. In addition, *C. carpio* has taken its place in the food groups since the age of 5. Tanyolaç and Karabatak [60] reported that wels catfish predated carp fish in Lake Mogan starting from the age of 5. It is thought that this is due to the fact that carp fish have higher body heights than other forage fish.

### 4.3. Feeding Features According to Length Groups

Fish constituted the most consumed food type in the diet of individuals belonging to the small, medium, and large-size groups. Benthic invertebrates were consumed only by small- and medium-sized individuals. As the size of wels catfish increased, the body height of the food intake also increased. So much so that carp fish, which were never included in the diet of small-sized individuals, partially appeared in medium-sized individuals, and their importance in the diet of large-sized individuals increased. Wysujack and Wysujack and Mehner [39] reported that as the size of wels catfish in Lake Feldberger Haussee increases, the size of food consumed also increases, and that the importance of *Perca fluviatilis* individuals in the diet, which have a larger body length compared to other fish. Carol et al. [8] reported that wels catfish up to 30 cm in Flix and Riba-roja reservoirs feed mostly on benthic invertebrates and plant materials, and then show an ontogenetic feeding feature, with freshwater crayfish as their main food. The same researchers also stated that the ontogenetic change was in fish in the Susqueda and Sau reservoirs. Pavlović et al. [51] determined that benthic invertebrates were consumed in individuals smaller than 60 cm in length, whereas they were not consumed at all in individuals larger than 60 cm, and the importance of fish in the diet increased even more. Didenko and Gurbyk [58] reported that as the size of wels catfish increases in the Kaniv reservoir, the importance of fish in the diet increases and the importance of invertebrates decreases. In addition, the same researchers reported that as the size of wels catfish increased, the size of the fish consumed also increased. Looking at the existing literature, it is seen that there are ontogenetic changes in the diet of wels catfish, and as the size of wels catfish increases, the size of the forage fish they feed on also increases. These two main results coincide with the findings of this study. On the other hand, the faunistic features of the habitats and the abundance of fish affect the main food consumed and ontogenetic nutritional change. For this reason, it is thought that it will not be possible to make comparisons between habitats based on these criteria.

### 4.4. Food Preference of Wels Catfish

According to Pearre's selectivity index values, small-sized fish preferred *A. boyeri*, while medium and large-sized fish preferred *T. tinca*. While medium-sized individuals avoided consuming *C. carpio*, *A. boyeri*, *S. glanis*, and *S. seyhanensis* species, they positively selected *T. tinca* species. It is thought that the negative selection of carp fish is due to their body height or the presence of spiny rays on their dorsal fins. In large-sized wels catfish, *A. boyeri* and *C. carpio* species were negatively selected, whereas *T. tinca* was positively selected and consumed. Negative selection of carp fish is not statistically significant. In this case, it can be said that the increase in the length of wels catfish increases the selectivity as well as the importance of species in the diet, especially those with relatively large body lengths, such as carp. In their experimental study, Adámek et al. [61] found that wels catfish showed negative selection towards *Rutilus rutilus* and *Pseudorasbora parva* species, slightly avoided consuming *Carassius auratus gibelio*, *Leuciscus cephalus*, and *Aspius aspius* species, and positively selected *Leucaspius delineatus*, *Scardinius erythrophthalmus*, and *Rhodeus sericeus*. Also, they reported that wels catfish mostly preferred to eat small-sized foods. Zaikov et al. [62], in their research conducted under controlled conditions, found that *S. glanis* individuals positively selected *P. parva* species in their feeding activities, while they negatively selected carp fish. On the other hand, there are also studies where wels catfish do not show any food preferences [40].

In conclusion, this study provides a comprehensive view of how the feeding habits of wels catfish (*S. glanis*) vary according to seasonal changes, age groups, and individual sizes. These findings allow us to understand how this species adapts to its environment and biological conditions and modifies its feeding strategies. The feeding habits of wels catfish offer critical insights for understanding the species' role in the ecosystem. Its opportunistic and diverse feeding behavior enables this species to adapt to different ecological conditions, which in turn affects population dynamics. From a management perspective, these findings provide important information for habitat management and fishing regulations for the wels catfish. For instance, considering the increased feeding activities during the breeding season and Winter months, it might be advisable to reduce fishing pressure during these periods. Additionally, the feeding preferences of different age and size groups are crucial factors to consider for population health and sustainable fishing practices. These details help us better understand the behaviour and feeding habits of wels catfish in their natural habitats. Furthermore, the applicability of this information in fisheries management and conservation strategies can contribute to the balance and sustainability of the species within the ecosystem.

## Figures and Tables

**Table 1:** The food composition of the *S. glanis*.

Prey	*N*	*N*%	*W*	*W*%	*F*	FO%	IRI	IRI%
Fishes								
* T. tinca*	144	52.5	724.77	37.3	50	47.7	4239.457	78.933
* C. carpio*	6	2.2	375.85	19.3	7	6.6	142.271	2.648
* A. boyeri*	48	17.5	38.82	2.0	17	16.01	313.012	5.828
* A. escherichii*	1	0.4	14.85	0.8	1	0.94	1.065	0.02
* Squalius seyhanensis*	3	1.1	12.55	0.6	2	1.9	3.285	0.062
* S. glanis*	1	0.4	0.06	0.003	1	0.94	0.347	0.005
Unidentified fish	23	8.3	41.66	2.2	14	13.2	139.199	2.592
Fish remains	—	—	191.46	9.9	40	37.7	372.043	6.926
Amphibia								
Ranidae	6	2.2	339.19	15.3	5	4.7	82.472	1.536
Crustacean								
* Potamon spp*.	4	1.5	48.1	2.5	4	3.8	14.855	0.277
* Astacus spp*.	7	2.6	69.85	3.6	6	5.6	34.820	0.649
Mammalia								
Rodentia	2	0.7	124.9	6.4	2	1.9	13.512	0.252
Mollusca								
Bivalvia	1	0.4	0.1	0.005	1	0.94	0.349	0.006
Benthic invertebrate								
Odonata	1	0.4	0.74	0.03	1	0.94	0.380	0.007
Isopoda	5	1.8	0.22	0.011	2	1.9	3.464	0.065
* Gammarus spp*.	8	2.9	0.84	0.043	2	1.9	5.590	0.104
Diptera pupae	3	1.1	0.06	0.003	1	0.94	1.035	0.019
Diptera larvae	11	4.0	0.11	0.005	1	0.94	3.792	0.071
Total	274	100	1941.95	100	157	—	5370.958	100
Number of full stomachs		106	—	—	—	—	—	—
Number of empty stomachs		94	—	—	—	—	—	—
Total number of stomachs		200	—	—	—	—	—	—

**Table 2:** Seasonal food composition in the wels catfish.

Prey	Seasons															
	Sprinng				Summer				Autmn				Winter			
	*N*%	*W*%	FO%	IRI%	*N*%	*W*%	FO%	IRI%	*N*%	*W*%	FO%	IRI%	*N*%	*W*%	FO%	IRI%
*T.tinca*	57.9	20.8	42.3	67.4	41.14	46.0	46.0	75.84	74.4	55.7	58.3	83.9	71.5	48.2	33.3	63.0
*C.carpio*	2.6	43.1	7.7	7.1	1.42	4.54	2.0	0.23	2.3	0	4.2	0.2	7.1	1.5	16.7	2.3
*A. boyeri*	15.8	1.8	15.4	5.5	25.53	3.51	26.0	14.3	0	0	0	0	0	0	0	0
*A. escherichii*	0	0	0	0	0.71	2.11	2.0	0.11	0	0	0	0	0	0	0	0
*S.seyhanensis*	0	0	0	0	2.84	1.76	4.0	0.29	0	0	0	0	0	0	0	0
*S.glanis*	0	0	0	0	0.71	0.008	2.0	0.028	0	0	0	0	0	0	0	0
Ranidae	5.3	20.6	11.5	6.1	1.41	19.34	4.0	1.57	0	0	0	0	0	0	0	0
*Potamon spp*.	0	0	0	0	2.84	6.77	8.0	1.46	0	0	0	0	0	0	0	0
*Astacus spp*.	2.6	2.9	3.8	0.4	3.55	6.73	10.0	1.94	0	0	0	0	0	0	0	0
Rodentia	0	0	0	0	0	0	0	0	2.3	21	4.2	1.1	7.1	33.6	16.7	10.7
Bivalvia	0	0	0	0	0.71	0.014	2.0	0.03	0	0	0	0	0	0	0	0
Odonata	0	0	0	0	0.71	0.104	2.0	0.032	0	0	0	0	0	0	0	0
Isopoda	6.6	0.03	7.7	1.1	0	0	0	0	0	0	0	0	0	0	0	0
*Gammarus spp*.	0	0	0	0	5.67	0.11	4.0	0.44	0	0	0	0	0	0	0	0
Dipter pupa	0	0	0	0	2.12	0.008	2.0	0.08	0	0	0	0	0	0	0	0
Dipter larva	0	0	0	0	7.81	0.016	2.0	0.3	0	0	0	0	0	0	0	0
Unidentified Fish	9.2	1.64	11.5	2.5	3.55	1.3	8.0	0.73	21	7	20.8	6.4	14.3	1.7	33.3	8.4
Fish Remains	0	9.13	53.8	9.9	0	7.68	18.0	2.62	0	14	54.1	8.4	0	15	66.7	15.6
Total	100	100	—	100	100	100	—	100	100	100	—	100	100	100	—	100
Number of full stomachs		26	—	—	—	50	—	—	—	24	—	—	—	6	—	—
Number of empty stomachs		23	—	—	—	48	—	—	—	22	—	—	—	1	—	—
Total number of stomachs		49	—	—	—	98	—	—	—	46	—	—	—	7	—	—

*Abbreviations:* A. boyeri, Atherina boyeri; A. escherichii, Alburnus escherichii; C. carpio, Cyprinus carpio; S. glanis, Silurus glanis; S. seyhanensis, Squalius seyhanensis; T. tinca, Tinca tinca.

**Table 3:** Feeding similarity between seasons in *S. glanis* individuals.

*C*_xy_	Spring	Summer	Autmn	Winter
Spring	—	—	—	—
Summer	**0.802165*⁣*^*∗*^**	—	—	—
Autmn	**0.785802*⁣*^*∗*^**	**0.629474*⁣*^*∗*^**	—	—
Winter	**0.755639*⁣*^*∗*^**	0.596251	**0.936877*⁣*^*∗*^**	—

*Note:* Bold values indicate that feeding activities are similar seasonally.

*⁣* ^*∗*^Statistically significant.

**Table 4:** Relative importance index values (IRI%) of food types according to age groups.

Prey	Ages										
	1	2	3	4	5	6	7	8	9	10	11
*T. tinca*	0	0	0	91.3	80.4	85.9	47.2	28.1	81.1	80.2	53.8
*C. carpio*	0	0	0	0	0.5	1	0	0	0	2.7	25.8
*A. boyeri*	75.2	90	97.6	2.8	4.91	0.6	10	4.3	0	3.8	0
*A. escherichii*	0	0	0	0	0	0.5	0	0	0	0	0
*S. seyhanensis*	0	0	0	0	0.09	0.4	0	0	0	0	0
*S. glanis*	0	0	0	0.1	0	0	0	0	0	0	0
Ranidae	0	0	0	0	2.9	0.7	0	0	0	10.7	13.7
*Potamon spp*.	0	0	0	0.5	0.71	0	0	7.4	0	0	0
*Astacus spp*.	0	0	0	2.5	1.42	0	0	6.8	0	0	0
Rodentia	0	0	0	0	0	0	9.1	26.8	0	0	0
Bivalvia	2.1	0	0	0	0	0	0	0	0	0	0
Odonata	0	0	0	0	0.06	0	0	0	0	0	0
Isopoda	0	0	0	0	0.6	0	0	0	0	0	0
*Gammarus spp*.	0	0	2.4	0	0.41	0	0	0	0	0	0
Dipter pupae	5.0	0	0	0	0	0	0	0	0	0	0
Dipter larvae	17.7	0	0	0	0	0	0	0	0	0	0
Unidentified fish	0	0	0	0.9	0.6	4.7	9.9	9.1	9.6	2.2	3.8
Fish remains	0	10	0	1.9	7.4	6.2	23.8	17.5	9.3	0.4	2.9
Number of full stomachs	2	2	1	19	41	19	8	6	2	3	3
Number of empty stomachs	1	1	3	19	40	18	1	8	1	0	2
Total number of stomachs	3	3	4	38	81	37	9	14	3	3	5

**Table 5:** Relative importance index values (IRI%) of food types according to length groups.

Prey	Length groups		
	20.1–47.0 cm	47.1–74.0 cm	74.1–101.0 cm
*T. tinca*	15.7	83.8	68.2
*C. carpio*	0	0.3	13.6
*A. boyeri*	73.3	2.3	2.6
*A. escherichii*	0	0.3	0
*S. seyhanensis*	0	0.1	0
*S. glanis*	0	0.009	0
Ranidae	0	0.871	7.3
*Potamon spp*.	0	0.4	0
*Astacus spp*.	2.5	0.8	0
Rodentia	0	0.07	1.8
Bivalvia	0.3	0	0
Odonata	0	0.011	0
Isopoda	0	0.09	0
*Gammarus spp*.	0.4	0.06	0
Dipter pupae	1.1	0	0
Dipter larvae	3.9	0	0
Unidentified fish	0.7	2.3	4.2
Fish remains	2.1	8.6	2.3
Number of full stomachs	12	84	10
Number of empty stomachs	14	72	8
Total number of stomachs	26	156	18

**Table 6:** Feeding similarity between length groups.

*C*_xy (cm)_	20.41–47.0 cm	47.1–74.0 cm	74.1–101.0 cm
20.1–47.0	—	—	—
47.1–74.0	0.5378	—	—
74.1–101.0	0.4406	**0.8534*⁣*^*∗*^**	—

*Note:* Bold value indicates that feeding activities of length groups are similar.

*⁣* ^*∗*^Statistically significant.

**Table 7:** Foods consumed by wels catfish indifferent studies.

Bruyenko [44]	Orlova and Popova [45]	Mukhamediyeva and Sal'nikov [46]	Bekbergenov and Sagitov [47]	Omarov and Popova [48]	Stolyarov [55]	Orlova and Popova [49]	Pouyet [56]	Mamedov and Abbasov [57]	
*A. brama*	*Alosa sp*.	*C. carpio*	*B. brachyceph*.	*C. carassius*	*A. gueldenstaedtii*	*Alosa sp*.	*A. brama*	*A. boyeri*	
*A. alburnus*	*A. brama*	*C. carassius*	*C. kuschkewits*.	*V. vimba persa*	*A. stellatus*	*Cobitis sp*.	*A. alburnus*	*Alosa sp*.	
*A. aspius*	*B. bjoerkna*	*Hemiculter sp*.	*R. rutilus*	*E. lucius*	*H. huso*	*C. carpio*	*B. bjoerkna*	*Cobitis sp*.	
*A. bipunctatus*	*P. cultratus*			*P. platygaster*	*A. boyeri*	*T. tinca*	*G. gobio*	*S. erythrophth*.	
*B. bjoerkna*	*S. lucioperca*			*P. fluviatilis*	*C. delicatula*	*E. lucius*	*R. amarus*	*A. alburnus*	
*C. carassius*	*S. glanis*			*R. frisii kutum*	*Cobitis sp*.	*P. platygaster*	*S. erythrophth*.	*A. aspius*	
*R. rutilus*	*A. aspius*				*A. brama*	*Neogobius sp*.	*L. gibbosus*	*B. brachyceph*.	
*V. vimba*					*C. carpio*	*S. lucioperca*	*P. fluviatilis*	*B. bjoerkna*	
*S. erythrophth*.					*R. rutilus*		*O. myksis*	*C. chalcoides*	
*E. lucius*					*S. erythrophth*.		*A. melas*	*C. carpio*	
*G. aculeatus*					*Neogobius sp*.			*R. amarus*	
*L. gibbosus*					*P. fluviatilis*			*R. rutilus*	
*Neogobius sp*.					*S. lucioperca*			*A.brama*	
*G. cernuus*					*S. nigrolineatus*			*P. platygaster*	
*P. fluviatilis*								*Neogobius sp*.	
*S. lucioperca*								*S. lucioperca*	
*S. glanis*								*C. wagneri*	
*C. taenia*								*S. glanis*	
*B. borysthenicus*									
*M. fossilis*									
*N. ophidion*									

Rossi et al. [50]	Czarnecki et al. ([54]	Doğan Bora and Gül [38]	Wysujack and Mehner [39]	Carol et al. [8]	Pavlović et al. [51]	Didenko and Gurbyk [58]	Didenko ve Gurbyk [58] Contiuned	Alp [40]	This Study
*A. alburnus*	*A. alburnus*	*T. tinca*	*A. anguilla*	*A. alburnus*	*A. alburnus*	*R. rutilus*	*G. cernuus*	*A. kotschyi*	*T. tinca*
*C. carassius*	*R. rutilus*	*S. lucioperca*	*A. brama*	*C. carpio*	*R. rutilus*	*S. erythrophth*.	*S. abaster*	*C. angorae*	*A. boyeri*
*L. cephalus*	*G. cernuus*	*S. glanis*	*A. bjoerkna*	*Liza sp*.	*P. fluviatilis*	*A. brama*	Frog	*C. erhani*	*A. escheric*.
*R. aula*	*P. fluviatilis*	*Diptera*	*S. erythrophth*.	*L. graellsii*		*B. bjoerkna*	*A. leptodactylus*	*C. carpio*	*C. carpio*
*C. soetta*	Duck	*Odonata*	*P. fluviatilis*	*R. rutilus*		*A. alburnus*	Spider	*L. pectoralis*	*S. galnis*
*P. flesus*	*A. leptodactylus*	*Gammarus*	*S. lucioperca*	*E. lucius*		*R. amarus*	Odanata.	*S. glanis*	*S. seyhan*.
		*Gastropoda*	*G. cernuus*	Birds		*T. tinca*	*A. aestivalis*	Crabs	Frog
		*Homoptera*	*R. rutilus*	*P. clarkii*		*C. gibelio*	Coleoptera	Leech	Crabs
		*Caryophyllaidae*	Birds	*G. holbrooki*		*C. taenia*	Coleoptera		Crayfish
			Mussel			*M. fossilis*	*D. polymorpha*		Rodent
						*N. fluviatilis*	*A. cygena*		Mussel
						*N. melanostomus*	*Viviparaus sp*.		Odonata
						*N. kessleri*	*P. corneus*		İsopoda
						*N.gymnotrachelus*	Plants		Gammarus
						*P. semilunaris*			Dipter pupae
						*P. glenii*			Dipter larvae
						*P. fluviatilis*			
						*S. lucioperca*			

*Note: Abramis bjoerkna*, *Abramis brama*, *Acipenser gueldenstaedtii*, *Acipenser stellatus*, *Alburnus escherichii*, *Alburnus alburnus*, *Alburnoides bipunctatus*, *Alburnus kotschy*, *Ameiurus melas*, *Anguilla anguilla*, *Anodonta cygnea*, *Aphelocheirus aestivalis*, *Aspius aspius*, *Astacus leptodactylus*, *Atherina boyeri*, *Barbus capito*, *Barbus lacerta*, *Barbus brachycephalus*, *Barbus borysthenicus, Blicca bjoerkna*, *Capoeta angorae*, *Capoeta capoeta*, *Capoeta erhani*, *Capoetabrama kuschkewitschi*, *Carassius gibelio*, *Carassius carassius*, *Chalcalburnus chalcoides*, *Chondrostoma oxyrhynchum*, *Chondrostoma soetta*, *Cyprinus carpio*, *Caspiomyzon wagneri*, *Clupeonella delicatula*, *Dreissena polymorpha*, *Gasterosteus aculeatus*, *Cobitis taenia*, *Gobio gobio*, *Gambusia holbrooki*, *Gymnocephalus cernuus*, *Esox lucius*, *Huso huso*, *Leuciscus cephalus*, *Lepomis gibbosus*, *Luciobarbus pectoralis*, *Luciobarbus graellsii*, *Misgurnus fossilis*, *Nerophis ophidion*, *Neogobius fluviatilis*, *Neogobius melanostomus*, *Neogobius kessleri*, *Neogobius gymnotrachhelus, Oncorhynchus myksis*, *Pungitius platygaster*, *Pelecus cultratus*, *Planobarius corneus*, *Platichthys flesus*, *Perca fluviatilis*, *Procambarus clarkii*, *Proterorhinus semilunaris*, *Percottus glenii*, *Rhodeus amarus*, *Rutilus aula*, *Rutilus frisii* kutum, *Rutilus rutilus*, *Sander lucioperca*, *Scardinius erythrophthalmus*, *Silurus glanis*, *Squalius seyhanensis*, *Syngnathus nigrolineatus*, *Syngnathus abaster*, *Vimba vimba*, *Vimba vimba* persa, *Tinca tinca*.

## Data Availability

The data for this article are available upon reasonable request to the corresponding author.
